# Construction and Use of Transposon *MycoTetOP*^2^ for Isolation of Conditional Mycobacteria Mutants

**DOI:** 10.3389/fmicb.2019.03091

**Published:** 2020-01-21

**Authors:** Sarah D. Riggs-Shute, Joseph O. Falkinham, Zhaomin Yang

**Affiliations:** ^1^Department of Biological Sciences, Virginia Tech, Blacksburg, VA, United States; ^2^Department of Biology, Tidewater Community College, Portsmouth, VA, United States

**Keywords:** transposon, mycobacterium, essential genes, conditional mutants, TetR-*tetO*

## Abstract

Mycobacteria are unique in many aspects of their biology. The development of genetic tools to identify genes critical for their growth by forward genetic analysis holds great promises to advance our understanding of their cellular, physiological and biochemical processes. Here we report the development of a novel transposon, *MycoTetOP*^2^, to aid the identification of such genes by direct transposon mutagenesis. This *mariner*-based transposon contains nested anhydrotetracycline (ATc)-inducible promoters to drive transcription outward from both of its ends. In addition, it includes the *Escherichia coli* R6Kγ origin to facilitate the identification of insertion sites. *MycoTetOP*^2^ was placed in a shuttle plasmid with a temperature-sensitive DNA replication origin in mycobacteria. This allows propagation of mycobacteria harboring the plasmid at a permissive temperature. The resulting population of cells can then be subjected to a temperature shift to select for transposon mutants. This transposon and its delivery system, once constructed, were tested in the fast-growing model *Mycobacterium smegmatis* and 13 mutants with ATc-dependent growth were isolated. The identification of the insertion sites in these mutants led to nine unique genetic loci with genes critical for essential processes in both *M. smegmatis* and *Mycobacterium tuberculosis*. These results demonstrate that *MycoTetOP*^2^ and its delivery vector provide valuable tools for the studies of mycobacteria by forward genetics.

## Introduction

The genus *Mycobacterium* contains *Mycobacterium tuberculosis*, the causative agent of tuberculosis (TB). As a major human pathogen, *M. tuberculosis* infects one third of the world population, resulting in 10 million cases of TB and approximately 1.6 million deaths per year (Bloom et al., 2017). Yet, many aspects of the unique biology of mycobacteria remain to be understood. One approach to study mycobacteria is to decipher the functions of core genes essential for their growth, and these genes may prove valuable for the understanding of mycobacterial biology as well as the research and development of antimycobacterial agents (Borsari et al., 2017; Singh and Mizrahi, 2017; Campanico et al., 2018; Evans and Mizrahi, 2018). However, experimental identification and validation of such genes are laborious because their null mutations lead to lethality. Instead, essential genes in mycobacteria are usually inferred statistically from the rarity of their mutants following high density transposon mutagenesis and deep sequencing of mutant pools (Sassetti and Rubin, 2003; Sassetti et al., 2003; Griffin et al., 2011; DeJesus et al., 2013, 2017). This approach has identified numerous genes that are likely essential for the viability of mycobacteria. On the other hand, only limited number of these genes have been studied functionally. The significant road block here is that mutants in these genes are not readily available for follow-up studies. Rather, their investigation requires reverse genetics to construct conditional mutants using regulated promoters to deplete a gene product (Schnappinger and Ehrt, 2014; Leotta et al., 2015; Boldrin et al., 2018, 2019).

A tetracycline regulatable system has been successfully adapted for use in mycobacteria for the construction of conditional mutations (Ehrt et al., 2005; Ehrt and Schnappinger, 2006). This system controls the expression of the tetracycline efflux pump TetA in *Escherichia coli* and other non-mycobacterial species. It is induced by tetracycline and its analogs such as anhydrotetracycline (ATc) with reduced antimicrobial activity or toxicity (Ehrt and Schnappinger, 2006). The minimum genetic determinants required for this regulation include the TetR repressor and its operator *tetO*. Ehrt and Schnappinger (2006) modified the promoter of the *rpsA* gene from *Mycobacterium smegmatis* (P_smyc_) by flanking its −35 sequence with two *tetO* operators. The resulting promoter, P_smyc1_*tetO*, was demonstrated to be induced by ATc once a *tetR* gene is introduced and expressed in *M. tuberculosis* and *M. smegmatis* (Ehrt and Schnappinger, 2006). Conditional mutants of essential genes were constructed using this promoter for both *M. smegmatis* and *Mycobacterium bovis* (Chalut et al., 2006), confirming the utility of this promoter for the regulation of essential genes in mycobacteria.

**FIGURE 1:**
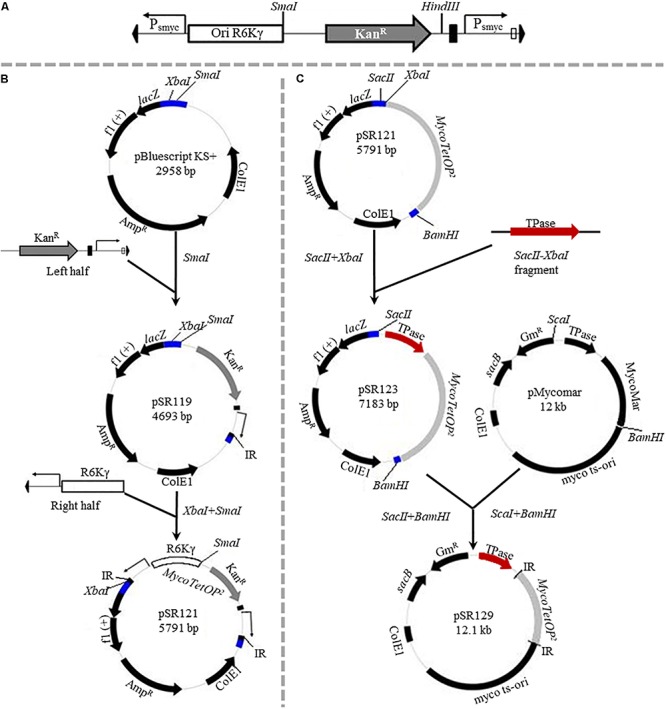
**(A)** Features of the *MycoTetOP*^2^ transposon. Drawn to approximate scale here is the *MycoTetOP*^2^ transposon of 2,851 base pairs. By design, it contains at its ends the two *mariner* IRs (block arrows in black), two P_smyc1_*tetO* promoters (labeled P_smyc_), the *E. coli* R6Kγ origin and the gene for Kan^*R*^. The direction of transcription from each promoter is indicated by the arrow above. A transcriptional terminator (T4g32T), shown as a solid rectangle in black, is placed downstream of the Kan^R^ gene. The *Mariner*_SP_R3 primer neighbors the right IR as indicated by the open rectangle. The transposon has unique recognition sites for *Sma*I and *Hin*dIII but none for *Sac*II. **(B)** Construction of *MycoTetOP*^2^. pSR119 and pSR120, containing the right and left halves of the transposon, respectively, were constructed as described in the text. The left half in a *Xba*I*-Sma*I fragment from pSR120 was cloned into similarly digested pSR119 to construct pSR121 with the complete *MycoTetOP*^2^. The region in blue is the multiple cloning site in pBluescript. **(C)** Construction of the delivery plasmid pSR129. A PCR fragment with the transposase (TPase) gene (in red) and pSR121 were both digested with *Sac*II and *Xba*I and ligated to generate pSR123. The TPase-*MycoTetOP*^2^ cassette in pSR123 was liberated by *Sac*II and *Bam*HI digestion and cloned into pMycoMar digested by *Sac*I and *Bam*HI as described in the text. The sizes of individual plasmids in this figure are indicated but they are not drawn to scale relative to one another.

The use of these regulated promoters in reverse genetics for the studies of essential genes is labor intensive, and there is a need for the development of tools for use in forward genetics. Herein are the results of the construction of a transposon with nested regulatable promoters and its introduction into *M. smegmatis* to generate conditional mutants by a forward genetic approach. The rationale was to place ATc-inducible and outward-facing promoters at both ends of and within a transposon. When the transposon inserts in either orientation near the 5′ end of an essential gene, the transposon mutant is expected to display inducer-dependent growth (Judson and Mekalanos, 2000). That is, such mutants may show normal or near normal growth with the inducer present while their mutant phenotypes can be analyzed in the absence of the inducer. In principle, this conditional phenotype can be used to screen for mutants to identify essential genes directly in a forward genetic screen. The new transposon, designated as *MycoTetOP*^2^ (Figure 1A), and its transposase gene were assembled and cloned into a delivery plasmid with a temperature-sensitive origin of replication in mycobacteria. A second plasmid, which integrates at a mycobacteriophage attachment site, was constructed to express TetR from a constitutive promoter. We tested *MycoTetOP*^2^ in a *tetR*-expressing *M. smegmatis* strain as a proof of principle. The results here validated the usefulness of *MycoTetOP*^2^ in the isolation of conditional mutants for the identification of essential genes in mycobacteria.

## Materials and Methods

### Bacterial Strains and Growth Conditions

All strains and plasmids used in this study are listed in Table 1. The *Escherichia coli* strains DH5αMCR (GibcoBRL) and DH5αλ*pir* were grown and maintained using Luria-Bertani (LB) media at 37°C (Miller, 1972). *M. smegmatis* strains were grown and maintained in LB liquid media supplemented with 0.05% Tween 80 or on LB agar at 30, 37 or 39°C as specified. All plates contained 1.5% agar and all liquid cultures were grown on a rotary shaker at 250 rpm.

**TABLE 1:** Bacterial strains and plasmids used in this study.

	**Descriptions**	**References**
**Strains**		
*E. coli*		
DH5αMCR	Cloning host	GibcoBRL
DH5αλpir	Cloning host, permissive for R6Kγ	Rubin et al., 1999
*M. smegmatis*		
mc2155	Electroporation-proficient variant of mc26	Snapper et al., 1990
VTY123	mc2155 transformed with pSR125	This study
**Plasmids:**		
pMycoMar	Tpase-magellan4, ts-ori, R6Kγ, KanR, GmR	Rubin et al., 1999
pMyr6k	pMycoMar parent, AmpR	Rubin et al., 1999
pMC1s	tetR, KanR, integrates at L5 attB	Ehrt et al., 2005
pSE100	T4 transcription terminator (T4g32T), HygR	Guo et al., 2007
pUV15tetORm	ATc-inducible promoter (Psmyc1tetO), HygR	Ehrt et al., 2005
pBluescript KS	Cloning vector, AmpR	Stratagene
pMe1ml1	Psmyc1tetO-lacZ reporter plasmid, HygR	Ehrt et al., 2005
pZerO-2	Cloning vector, KanR	Invitrogen
pSR114	*magelle4* in a pZero-2 derivative	This study
pSR117	P_smyc1_*_tetO_-lacZ*, pMe1mL1 with *tetR* removed	This study
pSR118	pSE100 with Hyg^R^ replaced by Kan^R^	This study
pSR119	Right half of *MycoTetOP*^2^ in pBluescript	This study
pSR120	Left half of *MycoTetOP*^2^ in pBluescript	This study
pSR121	Complete *MycoTetOP*^2^ in pBluescript	This study
pSR123	Tpase-*MycoTetOP*^2^ cassette in pBluescript	This study
pSR125	*tetR*, pMC1s with Kan^R^ replaced by Hyg^R^	This study
pSR126	P_smyc1_*_tetO_-lacZ*, pSR117 with Kan^R^ replacing Hyg^R^	This study
pSR129	Tpase-*MycoTetOP*^2^, ts-ori, R6Kγ, Kan^R^, Gm^R^	This study

When appropriate, ampicillin (Amp), kanamycin (Kan) and hygromycin (Hyg) at 100 μg/ml were used for selection in *E. coli*. For selection in *M. smegmatis*, growth media were supplemented with Kan and Hyg at 50 μg/ml or gentamycin (Gm) at 15 μg/ml. Anhydrotetracycline (ATc) at 50 ng/ml was used for the induction of the TetR-*tetO* regulated promoters in *M. smegmatis* as previously reported (Ehrt et al., 2005).

### Plasmid Construction

pSR114 was constructed as a PCR template for the amplification of the different elements in the *mariner*-based transposon *megallan4* (Rubin et al., 1999). Its backbone was a pZerO-2 derivative with an intact multiple cloning site (Table 1). An *Xba*I*-Spe*I fragment containing *megallan4* was obtained from pMyr6K (Rubin et al., 1999) and cloned into the *Xba*I and *Spe*I sites of this vector to generate pSR114.

pSR119, which contains the right half of *MycoTetOP*^2^ (Figure 1), was constructed as follows. A 1.2 kb fragment with the kanamycin resistance (Kan^R^) gene was PCR amplified (Black et al., 2010) from pZerO-2 with primers pZerO_kanR_F (CCCGGGCCCGGTACCGAGGACACGTAGAAAGCCAGTCC GCA) and pZerO_kanR_R (GCTAAGCTTGGAACAACACTCA ACCCTATCGC). This fragment was digested with *Hin*dIII and cloned into *Eco*RV and *Hin*dIII digested pSE100 (Guo et al., 2007) to generate pSR118; this placed T4g32T, a T4 phage transcription terminator, downstream of the Kan^R^ gene. Next, the P_smyc1_*tetO* promoter and a *mariner* IR were PCR amplified with the P_R1 (GGATCGTGCTCATTTCGGGC) and P_F1 (AATATTGGATCGTCGGCACC) primer pair and the M13F (CCCAGTCACGACGTTGTAAAACG) and 3_P_F2 (GCCCGAAATGAGCACGATCCTTCGACGCGTCAATTCGA GG) pair from pUV15*tetO*Rm and pSR114, respectively. These two fragments were joined to produce a P_smyc1_*tetO-mariner* IR fragment by overlapping PCR (Black et al., 2010) because of primer complementarity. Lastly, the Kan^R^-T4g32T cassette from pSR118 was PCR amplified using primers pZErO_kanR_F and T4g32_kanRterm_R (GGTGCCGACGATCCAATATTTAGCAATGCCTCCATGCG AT). This fragment was connected to the P_smyc1_*tetO-*IR fragment by overlapping PCR. The resulting PCR product, which contains all four elements for the right half of the designed transposon (Figure 1A), was cloned into *Sma*I-digested pBluescript KS to construct pSR119 (Figure 1B).

pSR120 contains the left half of *MycoTetOP*^2^ (Figure 1A). Three DNA fragments containing a *mariner* IR, the P_smyc1_*tetO* promoter and the R6Kγ replication origin were PCR amplified as follows. The IR and the R6Kγ fragments were PCR amplified from pSR114 using the M13R (AGCGGATAACAATTTCACACAGG) and 5_P_R1 (GCCCGA AATGAGCACGATCCTAATATGCATTTAATACTAG) primer pair and the P_F2 (GGTGCCGACGATCCAATATTTATATAGT ACCAACCTTCAA) and the P_R2 (TCCTCGGTACCG GGCCCGGGATCCCTTAATTAACCCCGAAAA) primer pair, respectively. The P_smyc1_*tetO* fragment was amplified from pUV15*tetO*Rm (Table 1) using primers P_R1 and P_F1. A joint IR-P_smyc1_*tetO* fragment was produced by overlapping PCR thanks to primer complementarity. This fragment was connected to the R6Kγ origin similarly by a second overlapping PCR. The resulting IR-P_smyc1_*tetO-*R6Kγ fragment was digested with *Xba*I and *Sma*I and cloned into the same sites of pBluescript KS to generate the plasmid pSR120 (Table 1).

pSR121 contains the fully assembled transposon. For its construction, the left half of *MycoTetOP*^2^ in pSR120 was liberated by *Xba*I and *Sma*I digestion and cloned into pSR119 digested by the same enzymes. Next, the transposase (Tpase) gene from pMycoMar (Rubin et al., 1999) was PCR amplified with primers TPase_F (TCCCCGCGGCCGTCCAGTCTGGCAG) and TPase_R (GCTCTAGATTATTCAACATAGTTCCCT), digested with *Sac*II and *Xba*I and cloned into pSR121 similarly digested. This gave rise to pSR123 which has the complete *MycoTetOP*^2^ transposon and its TPase gene (Tpase– *MycoTetOP*^2^) on a contiguous stretch of DNA in pBluescript with ampicillin resistance (Amp^R^) and the *E. coli* ColE1 replication origin (Figure 1C and Table 1).

pSR129 is an *E. coli*-mycobacterial shuttle plasmid with the TPase-*MycoTetOP*^2^ cassette (Figure 1C). For its construction, pSR123 was digested with *Sac*II, blunt-ended with T4 DNA polymerase and followed by digestion with *Bam*HI. The liberated *MycoTetOP*^2^-Tpase cassette was ligated with the backbone of pMycoMar (Rubin et al., 1999) obtained by digestion with *Sca*I, followed by blunt-ending with T4 DNA polymerase and digestion with *Bam*HI. This produced the shuttle plasmid pSR129 with the mycobacterial temperature sensitive replication origin (ts-ori) (Rubin et al., 1999) as well as the *E. coli* ColE1 origin and gentamycin resistance (Gm^R^).

The integrative plasmid pSR125 with the *tetR* gene was constructed from pMC1s (Guo et al., 2007) by replacing its Kan^R^ with Hyg resistance (Hyg^R^). First, the Kan^R^ gene in pMC1s was removed by *Nsi*I and *Bsp*HI digestion with a T4 DNA polymerase treatment in between to repair the overhand from *Nsi*I digestion. Next, a fragment carrying Hyg^R^ was obtained by digestion of pSE100 (Guo et al., 2007) with *Sma*I and *Bsp*HI. Ligation of the linearized pMC1s and the above fragment resulted in pSR125 which carries the *tetR* gene and Hyg^R^.

A new reporter plasmid, pSR126, was constructed for the analysis of P_smyc1_*tetO* regulation by TetR when it is expressed from pSR125 in *M. smegmatis. tetR* was removed from pMe1ml1 (Ehrt et al., 2005), which contains a P_smyc1_*tetO*-*lacZ* fusion, by *Not*I digesting and re-ligation to generate pSR117. The Hyg^R^ gene in pSR117 was removed by *Sac*II digestion and the backbone was treated with T4 DNA polymerase. The Kan^R^ region from pZerO-2 was PCR amplified with primers pZerO_kanR_F and pZerO_kanR_R and ligated with the backbone of pSR117 to generate pSR126.

### Transposon Mutagenesis of *M. smegmatis*

*Mycobacterium smegmatis* mc^2^155 was transformed by electroporation (Parish and Brown, 2008) with the *tetR*-expressing plasmid pSR125 to produce the strain VTY123 by Hyg^R^ selection. Next, the *MycoTetOP*^2^ delivery shuttle plasmid pSR129 was transformed into VTY123 by Gm^R^ selection. After a 4-hour recovery at 30°C in LB broth, cells were spread on LB plates containing Gm and incubated at 30°C for 4 days. An estimated 88,000 transformants were harvested from the selective medium and suspended at approximately 1.2 × 10^7^ cells per ml (cells/ml) in LB media with Tween 80 by passing through a syringe. This cell suspension was aliquoted and stored at −80°C in 15% glycerol as frozen stocks. To identify transposon mutants, approximately 1.2 × 10^4^ cells from the frozen stocks were spread on an LB agar plate (100 mm × 15 mm) with Kan and ATc. These plates were incubated for 3 days at 39°C, the non-permissive temperature for the ts-ori replication origin (Guilhot et al., 1992; Pelicic et al., 1997). Colonies that formed on these plates were presumed to be transposon mutants without the delivery plasmid.

### *MycoTetOP*^2^ Mutant Screening

Mutant colonies obtained in the presence of ATc at 39°C above were replica-plated onto medium with and without ATc and allowed to grow for 2 days at 39°C. Colonies that appeared to display ATc-dependent growth were patched in triplicates onto plates with and without ATc to verify the mutant phenotype after 2 days of incubation at 37°C besides testing for Gm^R^ for the loss of the plasmid. Mutants demonstrating reproducible ATc-dependent growth on patch plates were cultured in liquid media to exponential growth. These cells were diluted to 1 × 10^6^, 1 × 10^5^, and 1 × 10^4^ cells/ml. 10 μl of each dilution was spotted on LB plates with and without ATc. Growth was examined and documented after 2 days incubation at 37°C.

### Identification of Insertions in Transposon Mutants

Genomic DNA of *M. smegmatis* mutants (Parish and Brown, 2008) was digested with *Sac*II (Figure 1A) and treated with DNA ligase. The ligation mix was transformed into DH5αλ*pir* which allows the functioning of the R6Kγ origin of replication within the transposon. Recovered plasmids from the transformants were sequenced at the Genome Sequencing Center at Virginia Tech using primer *Mariner*_SP_R3 (TGAAGGGAACTATGTTGAAT) which recognizes the right end of *MycoTetOP*^2^ and read outwards from the transposon (Figure 1A). The sequences of the DNA flanking a *MycoTetOP*^2^ transposon insertion were analyzed using BLAST search against the genome sequences of *M. smegmatis* mc^2^155 at NCBI (Altschul et al., 1990; Mount, 2007) to identify the transposon insertions site. ORFs in the neighborhood of an insertion were searched against the complete genome sequence of *M. tuberculosis* H37Rv using BLAST tools (Altschul et al., 1990; Mount, 2007) and analyzed additionally using the MycoBrowser Portal (Kapopoulou et al., 2011).

## Results

### Design and Construction of *MycoTetOP*^2^ and Its Delivery Plasmid

*MycoTetOP*^2^ (Figure 1A), a *mariner*-based transposon, was designed for mutagenesis of essential genes in mycobacteria. The *mariner* element was chosen due to its relaxed site specificity for transposition and functionality in mycobacteria (Rubin et al., 1999). The starting template was *magellan4*, a *mariner* derivative that has been used successfully in mycobacteria (Rubin et al., 1999). *magellan4* contains the *E. coli* R6Kγ origin of replication and Kan^R^. One feature of the new transposon was the addition of the strong and inducible mycobacterial promoter P_smyc1_*tetO* nested inside the transposon and immediately adjacent to both IRs. P_smyc1_*tetO* is an engineered mycobacterial promoter that is repressed by TetR-*tetO* interaction and induced by ATc which binds and inactivates the TetR repressor (Ehrt et al., 2005). The two P_smyc1_*tetO* promoters (for the namesake of *TetOP*^2^) are positioned to drive transcription outward from *MycoTetOP*^2^ in both directions once it is transposed onto the mycobacterial chromosome. Another feature of *MycoTetOP*^2^ was the introduction of the transcription terminator from gene 32 of phage T4 (T4g32T) (Kaps et al., 2001; Guo et al., 2007) downstream of the Kan^R^ gene to prevent transcriptional read-through (Figure 1A). In principle, if *MycoTetOP*^2^ inserts in either orientation near the 5′ end of an essential gene, the transposon mutant may display ATc-dependent growth.

For the construction of *MycoTetOP*^2^, its left and right halves as defined by a *Sma*I site (Figure 1A) were assembled separately (see section “Materials and Methods”). The left half of *MycoTetOP*^2^ with the R6Kγ origin (Figure 1A) was assembled from three PCR fragments by two rounds of overlapping PCR and cloned into the *E. coli* vector pBluescript to generate pSR120 (Table 1). The right half of *MycoTetOP*^2^, assembled through a series of PCR reactions in combination with molecular cloning, was placed into pBluescript to construct pSR119 (Figure 1B). The last step was to join the left with the right at the *Sma*I site in pSR119 to assemble the full *MycoTetOP*^2^ transposon in pSR121 (Figure 1B).

pSR129 (Figure 1C), an *E. coli*-mycobacteria shuttle plasmid, was constructed for the delivery of *MycoTetOP*^2^ into mycobacteria. The *mariner* transposase (TPase) (Rubin et al., 1999) was first cloned next to the transposon to produce a Tpase-*MycoTetOP*^2^ cassette in the *E. coli* plasmid pSR123 (Figure 1C). Transposon mutagenesis of *M. smegmatis* with this plasmid by direct transformation was possible but inefficient (unpublished observations), likely because of the dual requirements for transformation AND transposition. The Tpase-*MycoTetOP*^2^ cassette was therefore combined with the backbone of pMycoMar which has ts-ori, a temperature sensitive mycobacterial replication origin to construct pSR129 (Guilhot et al., 1992). This shuttle plasmid can be transformed into mycobacteria by Gm^R^. Transformants can be propagated at the permissive temperature of 30°C and transposition can then be selected by Kan^R^ at the non-permissive temperature of 39°C.

### Identification of *M. smegmatis* Transposon Mutants With ATc-Dependent Growth

The use of *MycoTetOP*^2^ to generate conditional transposon mutants requires a strain that expresses TetR to regulate the P_smyc1_*tetO* promoters. Available was the plasmid pMC1s which can integrate at the phage L5 attachment site in mycobacteria to express TetR constitutively (Guo et al., 2007). However, this plasmid carries the same Kan^R^ selection as the *MycoTetOP*^2^ transposon. The Kan^R^ marker on pMC1s was thus replaced with a Hyg^R^ gene from pSE100 (Guo et al., 2007). The resulting plasmid, pSR125, was introduced by electroporation into the *M. smegmatis* strain mc^2^155 (Snapper et al., 1990) to construct the TetR-expressing strain VTY123. When VTY123 was transformed with the P_smyc1_*tetO*-*lacZ* reporter plasmid pSR126, the transformants exhibited ATc-inducible expression of β-galactosidase (data not shown) in line with previous observations (Ehrt et al., 2005).

The *MycoTetOP*^2^ delivery plasmid pSR129 (Figure 1C) was transformed into the TetR-expressing *M. smegmatis* strain VTY123 by Gm^R^ selection on plates at 30°C. After 4 days of incubation, an estimated 88,000 colonies were harvested. These cells were suspended and aliquoted for storage at −80°C for late use in mutant screening without further growth. This was intended to minimize the over representation of any cell with an early transposition event prior to screens for transposon mutants.

Next, a genetic screen was carried out to isolate *M. smegmatis* transposon mutants with ATc-dependent growth. Cells from frozen stocks above were plated to select for Kan^R^ colonies at 39°C (Guilhot et al., 1992) in the presence of ATc and ∼5,200 Kan^R^ colonies were screened by replica plating. 13 mutants, designated VTY201-213, were found to have ATc-dependent growth in primary screens. The conditional growth phenotype of these mutants was confirmed by patching in triplicates on plates with and without ATc. These mutants were all found to be sensitive to gentamycin because its resistance is carried by the delivery plasmid instead of the transposon itself.

**FIGURE 2:**
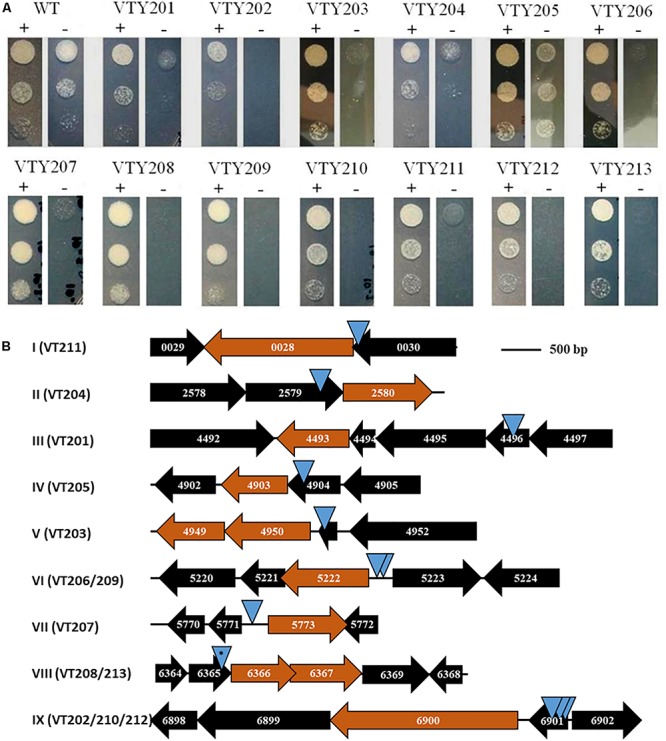
**(A)** ATc-dependent growth of mutants. The 13 mutants and their isogenic wild-type (WT) parent VT123 were examined for growth with (+) and without (–) ATc. 10 μl of cell suspension at 1 × 10^6^, 1 × 10^5^, and 1 × 10^4^ cells/ml of an indicated strain were spotted on plates shown here from top to bottom. Growth was examined after 2 days of incubation at 37°C. **(B)** Loci identified by *MycoTetOP*^2^ insertions. Drawn to approximate scale are the ORFs and their MSMEG numbers at the nine genetic loci (I–XI) identified by *M. smegmatis* conditional mutants. The candidate essential genes at each locus is colored in maroon. Each blue triangle represents a unique transposon insertion. Two identical insertions at locus VIII (^∗^) were found in two separate mutants (see text for details).

The growth of the 13 mutants was further examined on agar plates with and without ATc by serial dilutions (Figure 2A). As expected, the wild-type parental strain (VTY123) showed no difference in growth with and without ATc. In contrast, the 13 mutants all showed various degrees of ATc-dependent growth. Based on their growth phenotypes in this test, these mutants were divided into three groups. VTY205 displayed only a weak growth deficiency without ATc whereas VTY201 and VTY204 showed a moderate ATc-dependency (Figure 2A). The remaining 10 mutants belong to the last group with the strongest ATc-dependent growth. That is, they showed no or little growth in the absence of ATc but grew similarly as the wild type in the presence of ATc in this assay. These results indicated that the *MycoTetOP*^2^ transposon fulfilled its designed purpose for the isolation of conditional mutants of mycobacteria for the identification of possible essential genes by direct transposon mutagenesis.

### Identification of Essential Genes Near *MycoTetOP*^2^ Insertions in Conditional Mutants

The transposon and its flanking DNA from each mutant were cloned and sequenced to identify the site of *MycoTetOP*^2^ insertion. Plasmids from different colonies on the transformation plate for each mutant were isolated and examined by multiple restriction digestions. For each of the 13 mutants, the same digestion pattern was observed for these plasmids (data not shown), indicating that there is likely only one single transposon insertion in each mutant. Sequences of mycobacterial DNA flanking *MycoTetOP*^2^ insertions were used in BLAST searches against the genome sequence of *M. smegmatis* mc^2^155 to identify the transposon insertion sites. This led to nine distinct genetic loci, designated I through IX, according to their genome coordinates (Figure 2B). The three mutants with moderate (VTY204 and VTY201) and weak (VTY205) ATc-dependent growth led to loci II, III, and IV, respectively. The 10 mutants with strong ATc-dependent growth identified the remaining six loci, I and V-IX. Among these, loci VI and IX were identified by two (VTY206 and VTY209) and three (VTY202, VTY210, and VTY212) independent insertions, respectively. Locus VIII was identified by two identical insertions in VTY208 and VTY213, possibly due to the propagation of the same insertion mutant during the screening process. The other three loci, I, V and VII were identified each by single insertions in VTY211, VTY203 and VTY207, respectively.

The ATc-dependent growth of these mutants suggests that one or more genes adjacent to their transposon insertions are possibly essential for *M. smegmatis* growth. To identify these putative essential genes, the genomic contexts of the nine genetic loci were analyzed in relations to their respective insertions (Figure 2B). The putative essential genes at these loci may be divided into three groups with three members each based on their known or putative functions (Table 2). Genes at loci II, VII, and VIII form the first group with functions in the biosynthesis of mycolic acids (MAs) or arabinogalactans (AGs) which form the outer layers of mycobacterial cell wall (Jankute et al., 2015; Bhat et al., 2017). MSMEG_2580 at locus II (Figure 2B) encodes GcpE/IspG, an enzyme in the synthesis of isoprenoid, a key precursor for cellular components including those required for cell wall biosynthesis (Brown et al., 2010; Artsatbanov et al., 2012). MSMEG_5773 (locus VII) encodes DesA1, which is essential in *M. smegmatis* with the proposed function of introducing double bonds in MAs (Singh et al., 2016). MSMEG_6366 and MSMEG_6367 at locus VIII, encoding an RfbE-like ABC transporter and the GlfT1 glycosyl transferase, respectively, are both required for AG biosynthesis (Belanova et al., 2008; Dianiskova et al., 2011). The second group, which include loci I, IV, and IX, identified genes implicated in the biosynthesis of peptidoglycan (PG) which form the inner layer of mycobacterial cell wall. At locus I, MSMEG_0028, which encodes the Ser/Thr protein kinase PknB implicated in PG biosynthesis (Chawla et al., 2014; Turapov et al., 2018), are known to be essential in *M. smegmatis* and *M. tuberculosis* (Fernandez et al., 2006; Chawla et al., 2014). At locus IV, MSMEG_4903 encodes the MurI glutamate racemase, an enzyme essential for PG biosynthesis (Li et al., 2014). At locus IX, MSMEG_6900 or *ponA* encode PBP1, a bifunctional penicillin-binding protein with both transglycosylase and transpeptidase activities essential for PG biosynthesis in *M. smegmatis* (Hett et al., 2010; Kieser et al., 2015). The last group included loci III, V, and VI and they harbor genes critical for ribosomal functions and translation. MSMEG_4493 (locus III) encodes the rRNA-binding GTPase Era known to be essential for bacterial viability (Ji, 2016). The insertion at locus V occurred in MSMEG_4951, which encodes the ribosomal protein L31. MSMEG_4950 and MSMEG_4949 immediately downstream encodes the protein chain release factor PrfA (Rodnina, 2018) and its modifying enzyme HemK (Yang et al., 2004), respectively. The insertions at locus VI were adjacent to MSMEG_5222, which encodes YchF, a universally conserved GTPase with possible involvement in translation (Becker et al., 2012; Rosler et al., 2015). In summary, the candidate essential genes identified by the 13 *M. smegmatis* mutants are known or suspected to have function in MA, AG and PG biosynthesis as well as the information flow from mRNA to protein. These results indicate that the *MycoTetOP*^2^ transposon and its delivery vehicle constructed here are useful tools for the investigation of mycobacteria by forward genetics.

**TABLE 2:** Gene annotations in *M. smegmatis* and *M. tuberculosis.*

**Loci**	**Msm**^∗^	**Functional Description**	**Mtb**^#^	
I	0028	Ser/thr protein kinase, PG synthesis	*pknB* (Rv0014c)	**+**, Chawla et al., 2014
II	2580	Isoprenoid synthesis, MA synthesis	*ispG* (Rv2868c)	**+**, DeJesus et al., 2017
III	4493	GTP-binding protein, Translation	*era* (Rv2364c)	**+**, Sassetti et al., 2003
IV	4903	Glutamate racemase, PG synthesis	*murI* (Rv1338)	**+**, Morayya et al., 2015
V	4950	Release factor (RF), translation	*prfA* (Rv1299)	**+**, Sassetti et al., 2003
	4949	RF modification methylase	*hemK* (Rv1300)	**+**, Sassetti et al., 2003
VI	5222	Ribosome binding, translation	*ychF* (Rv1112)	**−**, DeJesus et al., 2017
VII	5773	fatty acid desaturase, MA synthesis	*desA1* (Rv0824c)	**+**, Sassetti et al., 2003
VIII	6366	ABC transporter, AG synthesis	*rfbE* (Rv3781)	**+**, Sassetti et al., 2003
	6367	Glycosyl transferase, AG synthesis	*glfT1* (Rv3782)	**+**, Sassetti et al., 2003
IX	6900	Penicillin-binding protein PBP1, PG synthesis	*ponA1* (Rv0050)	**+**, Hett et al., 2010

*^∗^ The *M. smegmatis* (Msm) genes inferred to function in essential processes at each locus is shown by their MSMEG ORF number. ^#^ The corresponding orthologous genes in *M. tuberculosis* H37Rv and their annotations are indicated in the first column under Mtb. In the next column, the + and – signs indicate the gene is essential and non-essential in *M. tuberculosis* H37Rv, respectively, with the relevant references.*

## Discussion

Here we describe the design, construction and use of a novel transposon, *MycoTetOP*^2^, for the identification of essential genes in *M. smegmatis* by transposon mutagenesis for the isolation of conditional mutants. We screened ∼5,200 *MycoTetOP*^2^ transposon insertions and identified 13 mutants with ATc-dependent growth phenotypes. These mutants led to nine genetic loci, each with candidate genes with functions in the essential processes of translation and the synthesis of mycobacterial cell wall (Figure 2 and Table 2). In principle, this transposon can be used in other mycobacteria including *M. tuberculosis* because both the *mariner* transposon and TetR-*tetO* regulatory systems have been demonstrated to function in other mycobacterial species (Rubin et al., 1999; Sassetti et al., 2001; Ehrt et al., 2005; Ehrt and Schnappinger, 2006; Guo et al., 2007).

Searches with *M. smegmatis* candidate genes at the nine loci against the genome sequence of *M. tuberculosis* indicated that they are all conserved in the TB strain (Table 2). All their *M. tuberculosis* counterparts, except YchF, are essential for viability of *M. tuberculosis* (Table 2). Some of these genes are known targets for antimycobacterial and/or antimicrobial agents. PknB, the ser/thr protein kinase critical for PG biosynthesis (Chawla et al., 2014; Turapov et al., 2018), has been explored as a target for antimycobacterial agents (Mori et al., 2019), for example. MurI (locus IV) is a glutamate racemase essential for PG biosynthesis in both *M. tuberculosis* and *M. smegmatis* (Li et al., 2014; Morayya et al., 2015) and it is a known target of anti-TB chemotherapeutics (Prosser et al., 2016; Pawar et al., 2019). PonA1 or PBP1 (locus IX) is a bifunctional enzyme with transglycosylase and transpeptidase activities required for PG synthesis and crosslinking (Barreteau et al., 2008; Vollmer and Bertsche, 2008; Hett et al., 2010; Kieser et al., 2015). It binds and is inhibited by β-lactams, the best known and the most important class of antibiotics in human history. There is renewed interest in PG biosynthesis as a target for anti-TB therapies as multidrug resistance becomes a serious threat (Catalao et al., 2019). In addition, GcpE/IspG (locus II) constitutes part of the methylerythritol phosphate (MEP) pathway which is considered promising targets for the development of antimycobacterial and antiparasitic agents (He et al., 2018; Wang and Dowd, 2018). The findings of these critical genes in this study illustrate the usefulness of our newly constructed transposon in the studies of mycobacteria.

Forti et al. (2011) constructed and applied a *marine*-based transposon with an outfacing promoter to identify *M. tuberculosis* conditional mutants. Their efforts produced 14 *M. tuberculosis* mutants, leading to the identification of 10 genes. The attempts to use their transposons in the fast-growing model mycobacterium *M. smegmatis* was unsuccessful and not further pursued. The design and construction of *MycoTetOP*^2^ occurred independently (Riggs, 2011) from the work by Forti et al. (2011), and there are a few differences in the designs and delivery of our transposon from theirs. First, *MycoTetOP*^2^ here uses the TetR-*tetO* regulated promoters instead of the *pip/pptr* system in theirs. Second, while their transposon has one outfacing promotor, ours has promoters at both ends which may improve the efficiency for isolation of conditional mutants. Third, our transposon contains the *E. coli R6K* replication origin which facilitates the cloning and identification of *MycoTetOP*^2^ insertions. Forth, we introduced a transcription terminator after the Kan^R^ gene to prevent possible read-throughs from its promoter and minimize unintended expression of mycobacterial genes in the absence of induction. Last but least, the delivery systems are different. Forti’s transposons were transduced into mycobacteria (Forti et al., 2011) while *MycoTetOP*^2^ here can be delivered by transformation using an *E. coli*-mycobacterial shuttle plasmid. The key difference here is that the temperature sensitive ts-ori origin in the *MycoTetOP*^2^ delivery plasmid separates the selection for transposition from the acquisition of cells with the transposon-Tpase DNA cassette. This may be the main reason why *MycoTetOP*^2^ succeeded in *M. smegmatis* while theirs did not. This is because in our case, cells with the transposon-Tpase DNA cassette can be propagated before the selection for transposition. It is noted that the genes identified by Forti et al. (2011) and those here have no overlaps. We suggest that it is advantageous to have different and new regulatory schemes (Schnappinger and Ehrt, 2014; Leotta et al., 2015; Boldrin et al., 2018, 2019) for the design of transposons for the investigation of mycobacterial biology in the future.

## Data Availability Statement

Publicly available datasets were analyzed in this study. This data can be found here: https://www.ncbi.nlm.nih.gov/search/all/?term=NC_008596.

## Author Contributions

SR-S and ZY conceived the project. SR-S performed the experiments with technical guidance from JF and ZY. All authors wrote the manuscript.

## Conflict of Interest

The authors declare that the research was conducted in the absence of any commercial or financial relationships that could be construed as a potential conflict of interest.
